# Dental erosion and erosive tooth wear in childhood and adolescence: from diagnosis to prevention

**DOI:** 10.1007/s40368-026-01233-8

**Published:** 2026-08-25

**Authors:** T. S. Carvalho, T. Baumann, S. H. Niemeyer, M. A. R. Buzalaf, P. Lingström, A. Lussi

**Affiliations:** 1 School of Dental Medicine, Department of Restorative, Preventive and Pediatric Dentistry, University of Bern, Bern, Switzerland; 2 Bauru School of Dentistry, University of São Paulo, Bauru, Brazil; 3 Institute of Odontology, Department of Cariology, University of Gothenburg, Gothenburg, Sweden; 4 University Hospital for Conservative Dentistry and Periodontology, Medical University of Innsbruck, Innsbruck, Austria; 5 University Medical Centre, Department of Operative Dentistry and Periodontology, University of Freiburg, Freiburg, Germany

**Keywords:** Erosion, Erosive tooth wear, Childhood, Adolescence, Prevention

## Abstract

**Background:**

Dental erosion and erosive tooth wear (ETW) are increasingly significant non-carious defects in industrialized countries. Reasons for an increased prevalence are mainly modern dietary habits, but an improved diagnostic awareness also contributes to an apparent increase in cases. Unlike caries, erosion involves acid-induced demineralization without bacterial involvement. When this is combined with mechanical wear, it results in ETW. Diagnosis commonly employs the Basic Erosive Wear Examination (BEWE), a standardized clinical index assessing lesion severity across sextants.

**Etiology and susceptibility factors:**

Primary teeth might be more susceptible to ETW due to thinner and less mineralized enamel with lower microhardness, and dentine with a higher carbonate content and higher tubule density. Longer acid exposures increase their demineralization rates compared to permanent teeth. The salivary pellicle offers variable protection, with children’s saliva showing distinct composition and efficacy. Key risk factors include frequent consumption of acidic beverages, certain medications, gastroesophageal reflux, and certain behavioral habits. Genetic, salivary, and lifestyle factors further modulate individual susceptibility.

**Prevention:**

Preventive strategies emphasize risk assessment, dietary modification, and chemical protection using different fluoride-based agents, which form acid-resistant surface layers reducing enamel and dentine solubility. Combinations with chitosan or other polymers may enhance protection. Novel approaches include bio-inspired and film-forming agents, pellicle modification with proteins (e.g., CaneCPI-5), and natural polyphenols that reinforce acid resistance and collagen stability. While laboratory and in situ studies support these strategies, long-term clinical validation remains limited.

**Conclusions:**

Comprehensive management of ETW should integrate early diagnosis, patient education, and multi-modal prevention, particularly targeting children and adolescents, where susceptibility and long-term consequences are greatest.

## Introduction

Dental erosion and erosive tooth wear (ETW) as forms of non-carious tooth substance defects have increasingly become more important in recent decades, especially in industrialized countries. In contrast to caries, dental erosion is defined as the demineralization of the tooth structure *without* the involvement of bacteria. Due to acid, the tooth structure softens (i.e., its hardness decreases). If there is a loss of tooth structure caused by abrasive processes of the softened surface, it is referred to as ETW. Since mechanical processes mostly due to tooth brushing – but also due to attrition, tongue movements and cheek contact – are unavoidable, the term erosive tooth wear (ETW) has generally become established. On the other hand, clinically relevant wear is hardly observed without prior softening (through erosion) of the tooth structure.

The prevalence of ETW in the general population is about 20–40% in the industrialized countries of the world (Schlueter and Luka 2018). For the primary dentition, the reported mean prevalence is 30–50%, with large difference between the continents as well as within single countries. For older children and adolescents with a mixed dentition, the prevalence is in the range of 20–40%, although most studies at this age range deal with the permanent teeth (Schlueter et al. 2025). Reasons for the increased prevalence of ETW in the recent decades include a changed lifestyle with more acidic food, increasing stress and the associated more acid regurgitation (reflux), but also better education and training of people in the field of oral health. The better knowledge of the professionals means that changes are noticed earlier and diagnosed more often.

Some wear during lifetime is physiological and normal. However, if the normal wear rate is already increased at a young age, long-term effects can impair the quality of life in adulthood. In certain groups of people, both the prevalence and severity of erosion and ETW can increase significantly. These include people with eating disorders in combination with vomiting, people with reflux and, above all, people with special diets or habits (Schlueter et al. 2025; Schlueter and Tveit 2014).

It is therefore very important that the population is educated and protected at an early stage, for example through adequate nutrition and oral hygiene with substances active against ETW. Diagnosis, recognition of risk factors, nutritional advice and prophylaxis with mouthwashes based on known ETW protective agents, such as fluoride-based agents, stannous ions (Sn^2+^), proteins or flavonoids, play or will possibly play an important role in the future. In consultations with professionals and patients, the question of the erosive potential of different drinks and foods comes up often, because they play an important role in the development of ETW that can be controlled by the patient.

There are considerable differences in the erosive potential of the substances tested. For example, there are acidic products that do not cause erosion and those with a higher pH value that have an erosive potential. There is a difference in sensitivity to dental erosion between permanent and primary teeth. These topics will be discussed in this article.

## Diagnosis

**Fig. 1:**
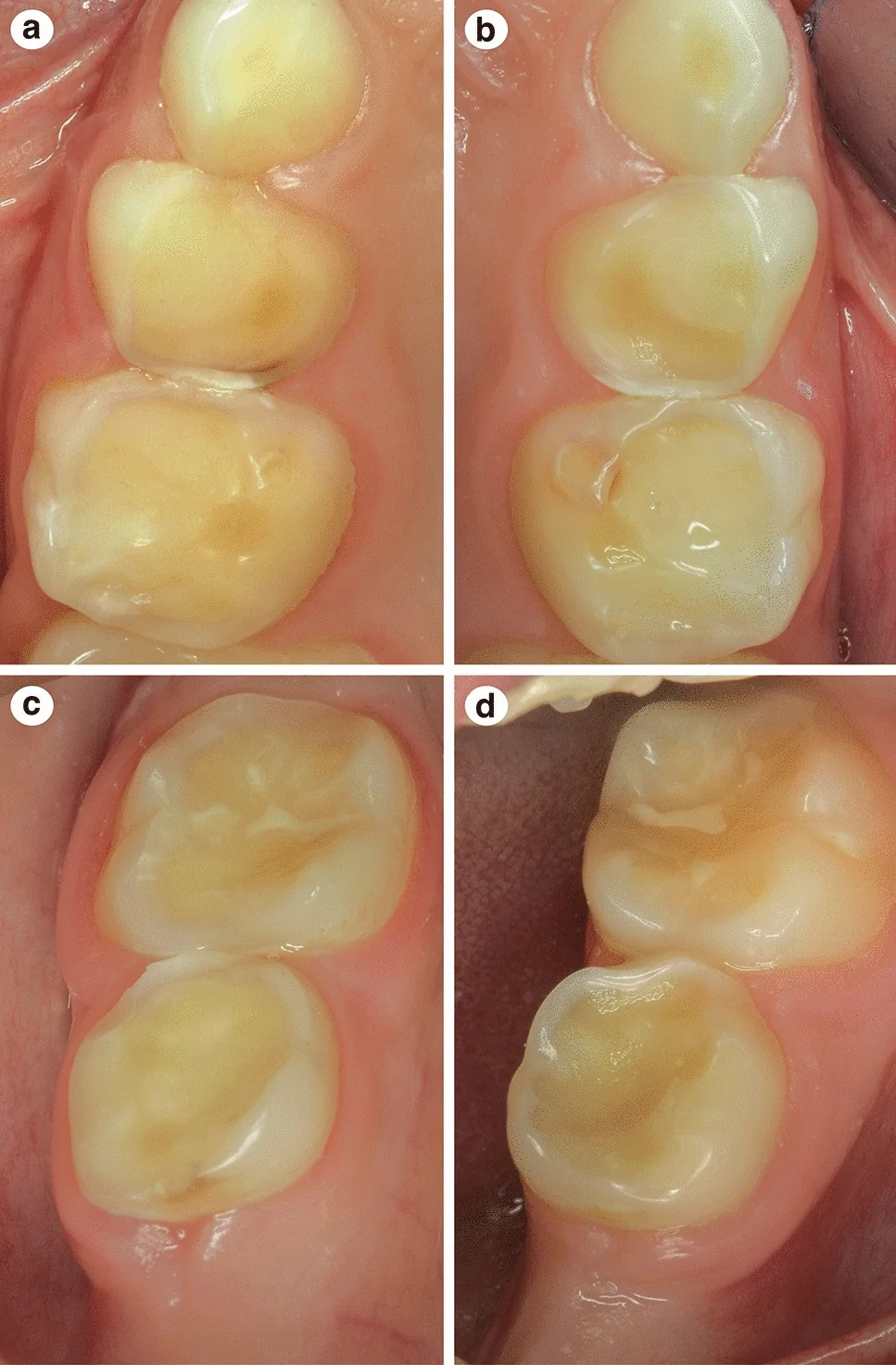
Upper (**a**, **b**) and lower (**c**, **d**) deciduous molars of a 12-year-old male. The physiological occlusal morphology is mostly worn off, and dentine is exposed (BEWE 3)

The diagnosis of ETW in children and adolescents (as well as adults) is primarily based on the clinical and visual assessment of hard dental tissues, combined with the identification of risk factors associated with the condition. In addition to the recognition of clinical features of ETW, photographic records, intraoral scans, and clinical indices may support the diagnostic process and are particularly valuable for monitoring the lesion over time. For assessment, the Basic Erosive Wear Examination (BEWE) is most often used (Bartlett et al. 2008). The BEWE was designed to avoid grading ETW based on exposed dentine. Although dentine is frequently exposed in stages 2 and 3 (fig. 1), BEWE does not include this factor in assessing the severity of the case because the irregularity of enamel thickness impedes direct correlation. For example, dentine is considerably more rapidly exposed at the neck of a tooth. Leaving out this factor eliminates a source of error and facilitates the comparison of data collected by different examiners. Moreover, the index can be recorded with the patient present or with models and photographic images. Further, it is an index that quantifies the size of a given lesion based on the percentage of the surface affected (table 1). It is easy to use and serves as the basis for recommendations regarding preventive and restorative measures (Bartlett et al. 2008). Except for the third molars, teeth are tested for vestibular, occlusal, and oral damage. In every sextant, the most severe score is recorded and a cumulative score from all sextants is calculated, which represents the index value. This procedure is also applied with children when some teeth may be missing.

**Table 1:** Basic erosive wear examination (BEWE) for assessing erosive tooth wear

Score	Criteria
0	No erosive tooth wear
1	Initial loss of surface texture
2	Distinct defect; hard tissue loss on <50% of the surface area*
3	Severe defect; hard tissue loss on ≥50% of the surface area*

*In scores 2 and 3, dentine is often involved, but dentine involvement is not used as a criterion for assessing the grade

While the original BEWE continues to be widely used in both research and clinical practice, a revised version, BEWE 2.0, has recently been introduced in an effort to improve its correspondence with clinical findings (Bartlett et al. 2026). Among the main changes are the inclusion of an additional severity level for more advanced wear and a revised approach to risk interpretation based on affected teeth rather than only on the cumulative sextant score. On the one hand, these modifications may favor its use in the primary dentition, in which the sextant-based risk assessment approach was less easily applicable and required substantial adaptation. But on the other hand, the newly proposed severity levels are conceptually unsound for deciduous teeth. Moreover, the interpretation of severity thresholds for deciduous teeth should still be made with caution, as wear may not be expressed in the same way as in permanent teeth.

The time interval recommended for assessments using the BEWE index depends on the severity of the erosive lesions, on individual risk factors and on the situation of the individual patient. Patients showing increased intrinsic and/or extrinsic acid exposure should be examined biannually, and the interval may be changed based on the risk for further progression (Carvalho et al. 2014)**.** As during childhood, the number of teeth is changing, the number of teeth per sextant also changes, which must be recorded. Otherwise, a longitudinal comparison of the data is not possible. This is important, considering that ETW in primary teeth is a predicting factor for ETW in permanent teeth.

## Susceptibility of primary and permanent teeth to ETW

During any clinical decision-making regarding ETW in paediatric dentistry, it is essential to understand the peculiarities of primary and permanent teeth, and how they react during erosive challenges. A growing body of research has focused on the structural and functional differences between these two types of dentition, as outlined below:

### Structural differences between primary and permanent teeth

The physical and chemical characteristics of primary and permanent teeth differ considerably and emphasise their respective susceptibilities to ETW. These structural differences are evident macroscopically as well as at a microscopic scale.

#### Enamel microstructure and properties

Primary teeth are generally smaller and markedly thinner (from outside to the dentine-enamel-junction—DEJ) than permanent teeth. They also have a lower mineral density than permanent enamel. Combined, these factors are probably responsible for their significantly lower surface microhardness (Johansson et al. 2001; Lippert et al. 2004; Lussi et al. 2000; Magalhães et al. 2009) and elasticity (Lippert et al. 2004). As a result, primary enamel can demineralize more rapidly and is less resistant to mechanical forces.

Both primary (Kodaka et al. 1989; Ripa 1966; Ripa et al. 1966) and permanent (Kodaka et al. 1991) enamel possess an aprismatic outer layer. The prisms in both kinds of enamel are organized in a similar orientation—typically perpendicular to the surface (Horsted et al. 1976)—but in primary enamel, the prisms are slightly more curved (Shellis 1984b), slightly more widely spaced out (Shellis 1984a), which may explain why they are more porous and, in turn, more soluble than permanent enamel.

Both types of enamel have a decreasing mineralization gradient, with greatest mineralization near the outer layers (superficial enamel) and decreasing values toward the DEJ. Despite this similarity, primary enamel is generally less mineralized (Wilson and Beynon 1989) than permanent enamel, and they also contain a higher proportion of carbonate ions (Sonju Clasen and Ruyter 1997) in their crystal structure. The carbonate ions substitute the hydroxide ions, forming type A carbonated hydroxyapatite. This type of hydroxyapatite crystal has a different lattice layout, leading to distortions in its structure and, thereby, increased enamel solubility.

#### Dentine differences

Fewer studies have focused on the comparison between primary and permanent dentine, but it is possible to outline some trends. Dentine in primary teeth have typically lower calcium-to-phosphorus ratios and greater carbonate content in their mineral structure (Torres et al. 2018). Another noteworthy histological difference is the higher tubule density in primary teeth (Lenzi et al. 2013), which may affect adhesion of restorative materials (de Los Angeles Moyaho-Bernal et al. 2018), but their effect on ETW is not yet known.

### Differences in susceptibility to ETW between primary and permanent teeth

Several studies have compared acid demineralization of primary and permanent teeth with conflicting results. Despite these differences, we can still reach some conclusions on the topic:

#### Influence of erosive exposure

Different studies have used different exposure times of the teeth to acidic substances. These different lengths of time lead to considerable differences in acid demineralization: in shorter exposure times (under five minutes), demineralization rates between primary and permanent teeth are similar, with no differences between both dentitions. In longer exposure times, however, the differences between primary and permanent teeth become much more apparent, where primary enamel end up demineralizing up to 1.5 times faster than their permanent counterpart (Featherstone and Mellberg 1981). This suggests that susceptibility to ETW may be “severity”-related or time-dependent. During occasional intake of acidic substances, similar demineralization will be observed on both kinds of teeth, but in more severe acid exposures, the relatively lower hardness and the different mineral structure of primary enamel will eventually lead to faster and greater demineralization.

#### The modulating role of the salivary pellicle

The salivary pellicle is a protein-rich layer that naturally forms on tooth surfaces when they are exposed to saliva. The pellicle serves as a semi-permeable layer, offering some protection against acidic attacks (Hannig et al. 2004; Hannig et al. 2003). In laboratory studies, where no saliva is used—therefore no pellicle is present—no significant differences are observed in the susceptibility of primary and permanent teeth to ETW. However, the differences become more evident in studies where the pellicle is present. Not only that, we (Carvalho et al. 2016) also showed that children’s saliva—children’s pellicle—seem to be more protective towards primary teeth, whereas adult saliva—adult pellicles—are more protective towards permanent teeth. This may be relevant during the mixed dentition phase, when the first permanent molars or the erupting premolars are still covered in children’s saliva—children’s pellicle. Notwithstanding these differences, the maturation of saliva during adolescence (salivary composition, pellicle proteomics, etc.) are factors that still remain to be fully understood.

### Clinical relevance

**Fig. 2:**
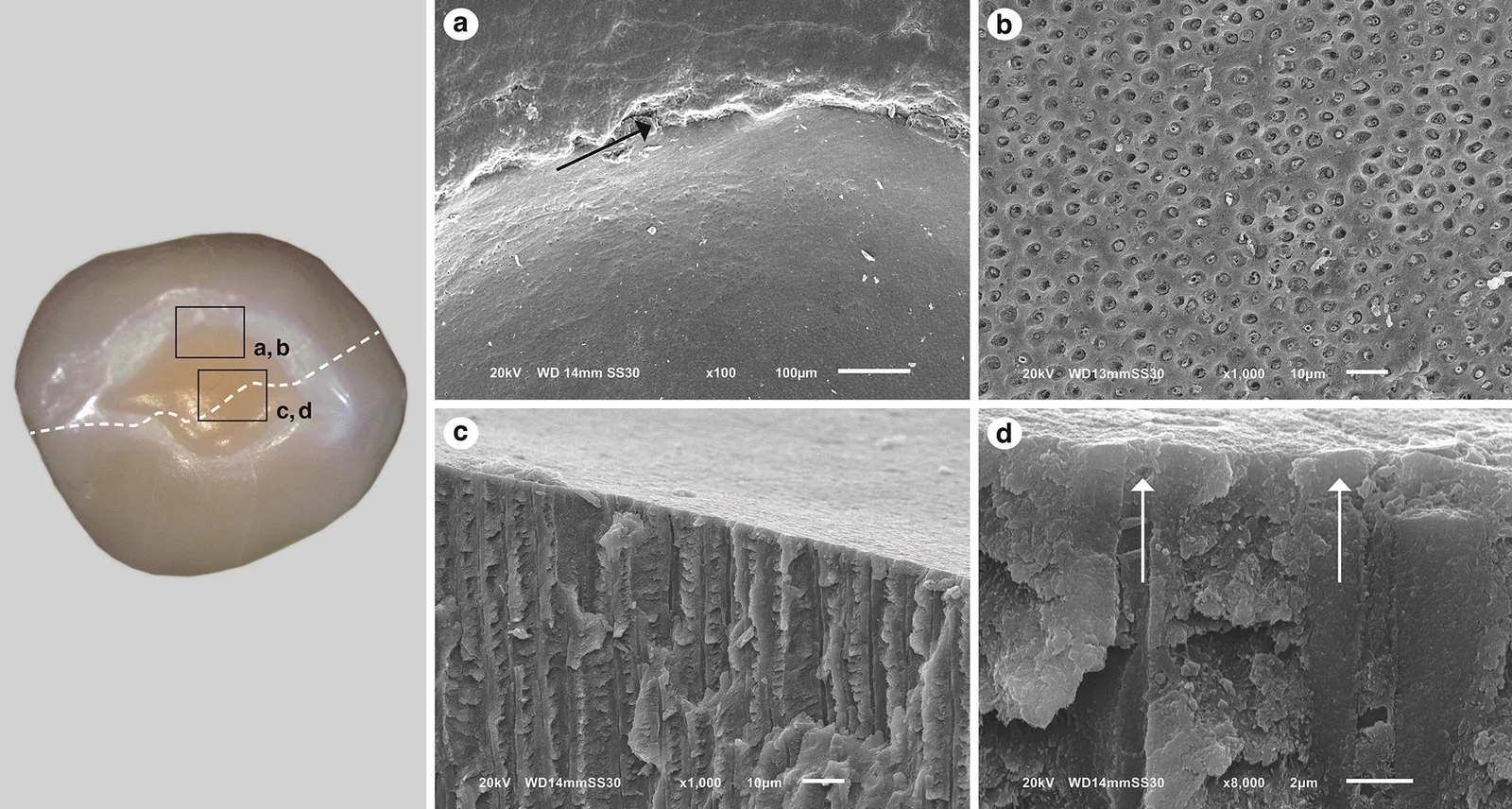
Deciduous canine showing an erosive tooth wear (ETW) lesion extending into dentine. The tooth was sectioned along the dashed line: (**a**,**b**) Scanning electron micrographs from the occlusal view; (**c**,**d**) from the sectioned surface. The black arrow (**a**) shows the enamel-dentine boarder; (**b**) shows the dentine surface with patent tubules; and the white arrows (**d**) show the opening of the dentine tubules towards the tooth surface. **a** ETW lesion at the interface (arrow) between enamel (upper) and dentine (lower). **b** Higher magnification of the dentine surface showing the open dentinal tubules. **c** Side view of the sectioned dentine illustrating dentinal tubule orientation. **d** Higher magnification of the tubules showing their openings (arrows) towards the dentine surface

Clinically, primary teeth seem to be more susceptible to ETW than permanent teeth (Kreulen et al. 2010). Despite being covered by children’s pellicle, it is probable that in high-risk patients or in those with more severe acidic intakes, the protective effect of the pellicle is diminished and the primary teeth are constantly under strain. For example, the frequent consumption of soft drinks can increase the susceptibility to ETW by 2.4 times (Li et al. 2012), and if acidic drinks are consumed at least 3 times a day, children have a higher probability to develop ETW (Murakami et al. 2011; O'Sullivan and Curzon 2000). In such case, the outer layers of enamel are demineralized and subsequently worn away from physical and mechanical processes like abrasion or attrition. The deeper layers of enamel then become exposed (fig. 2), and it is known that the rate of mineral loss increases towards the DEJ. Since enamel in primary teeth is thinner, dentine is soon exposed (Magalhães et al. 2009; Rios et al. 2007), and this cascading effect, together with the structural peculiarities of primary teeth, increases the overall rate of progression of ETW. In practice, paediatric dentists may therefore need to take a more proactive management when protecting children’s teeth against ETW.

## There is no fixed critical pH for ETW

Tooth erosion is the result of the demineralization of tooth mineral by erosive substances. The pH of these substances plays an important role, and it is probably the most important single factor in predicting their erosive potentials (Shellis et al. 2025). But the demineralization is not strictly governed by the pH, but actually by the saturation of a potentially erosive substance with respect to the tooth material of interest (enamel, dentine, or cementum). This saturation is calculated from the ion activity product (IAP) of the substance, which is dependent on its composition and hence also its pH, and the solubility product (Ks) of the tooth mineral, which is a constant at a given temperature. If IAP is smaller than Ks, a substance is undersaturated and it will cause demineralization, whereas if IAP is larger than Ks, the substance is oversaturated and will lead to deposition of mineral or crystal growth. Since the main mineral of all tooth materials is hydroxyapatite, which is made up of calcium, phosphate, and hxdroxyl ions, the concentrations of these ions are also the most important for the calculation of the saturation. Others also play a role, since the tooth minerals are not pure hydroxyapatite, but the hydroxyl ions are the ones that link the saturation of a substance to the pH, since the concentration of hydroxyl ions is inversely proportional to the pH.

Given the influence of the pH on the saturation, the concept of a critical pH (pHc) has been established for caries. The pHc is the pH at which a surrounding solution is saturated with respect to the mineral of interest, and a lower pH than that will lead to undersaturation, and hence demineralization (Dawes 2003). In the case of caries, the pHc has a fixed value of 5.5-5.7 for enamel (ten Cate 2003). This is only possible because caries develops in the somewhat protected environment of dental plaque, and the pHc refers to plaque fluid, which is the solution in direct contact with the tooth surface in that case. Plaque fluid has a rather constant composition in regard to calcium and phosphate, therefore the only variable is the hydroxyl ion concentration, and a specific “universal” and fixed pHc can be calculated. In case of erosion, the solutions in direct contact with the tooth surface are the erosive substances, and they vary widely in their composition, also in calcium and phosphate concentrations. Therefore, no fix pHc for erosion can be defined, but it has to be calculated for each substance based on its individual composition. Simplified, if the pH of the substance is then below its calculated pHc, the substance will be erosive and cause demineralization. And the larger the difference between the actual pH and the pHc, the higher is the erosive potential of the substance. Consequently, some substances with low pH are also not as erosive as other substances with higher pH (Lussi et al. 2019b), because their composition results in a low pHc.

Deciduous teeth might be more susceptible to erosion, as they are more soluble (Cutress 1972). This is at least partly a result of the different composition of deciduous and permanent enamel, which also leads to different solubility products for the two. As the solubility product is part of the calculation for the pHc, the pHc in respect to deciduous enamel will be different from the pHc in respect to permanent enamel. Actually, the pHc for deciduous enamel will be higher than the pHc for permanent enamel. This usually enlarges the difference of the actual pH of the product to its specific pHc, making most substances potentially more erosive toward deciduous teeth than toward permanent teeth. Still, deciduous teeth have not always been shown to be more susceptible to erosion than permanent teeth (Lippert et al. 2004; Magalhães et al. 2009) but this might be due to protocol designs (Carvalho et al. 2022). 

## Factors related to ETW in childhood and adolescence

**Fig. 3:**
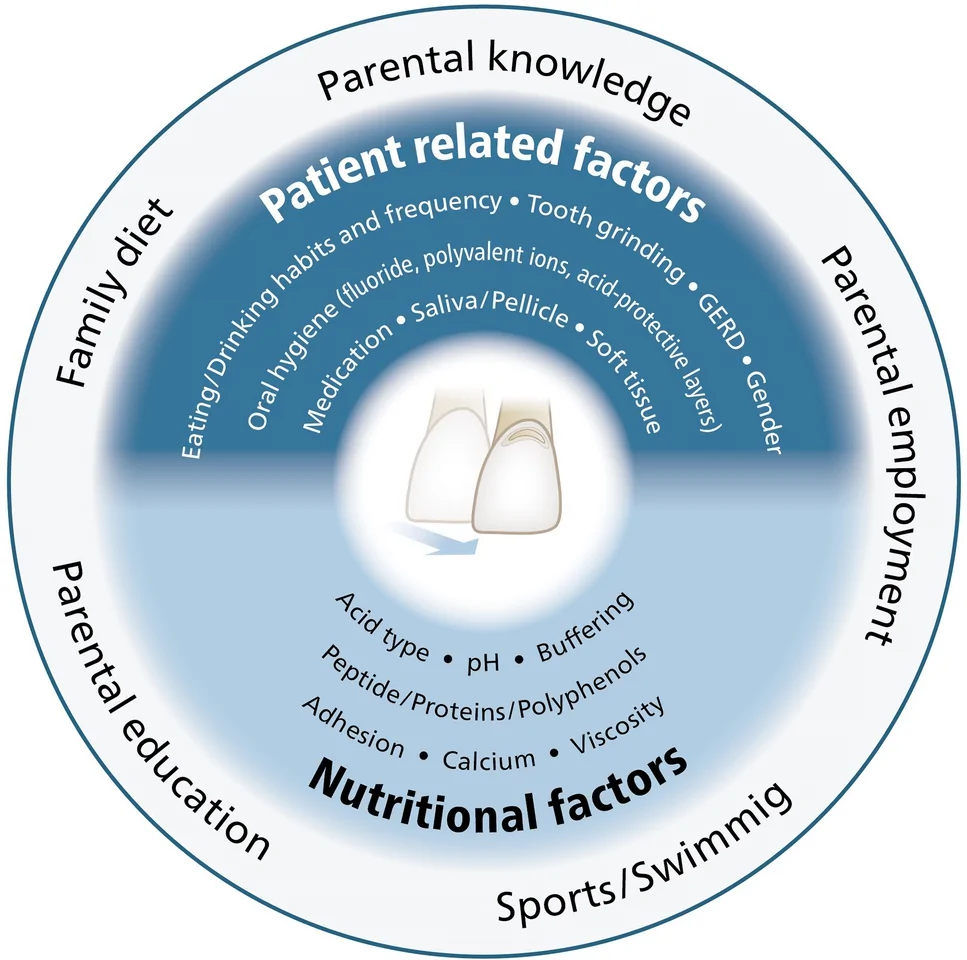
Factors related to ETW for children and adolescents

Factors related to ETW may be generally divided into two main groups: those related to nutrition and environment, and those related to the patients. In childhood and adolescence, these factors additionally have to be considered in context of family related factors (fig. 3). The main factors are summarized in table 2.

**Table 2:**
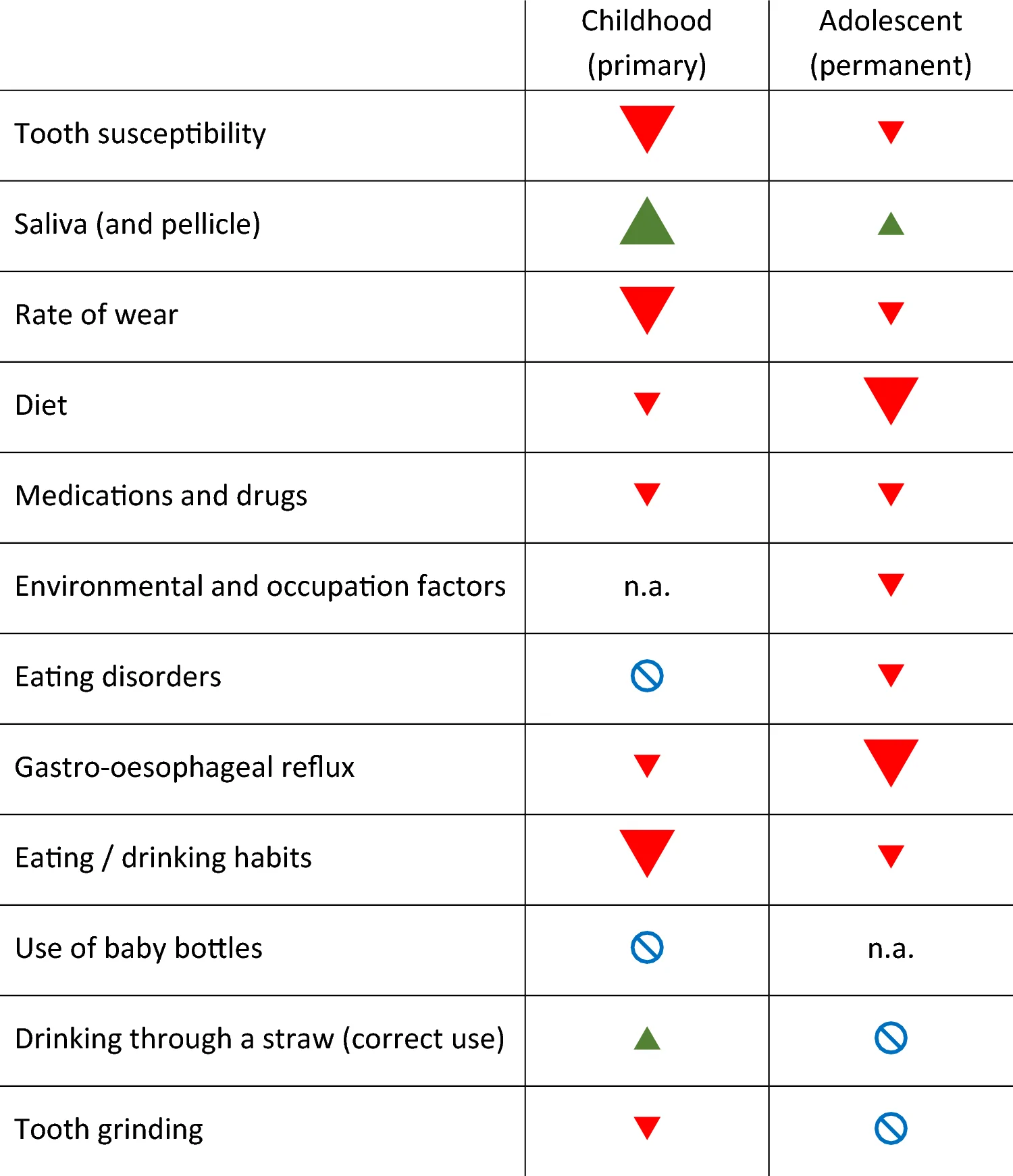
Summary of the factors related to ETW. The following classification system was applied, based on the evidence discussed and summarized in the text:  indicate associated factors,  indicate protective factors,  indicate lack of evidence, n.a. means not applicable, and the size of the arrow (small/big) indicate minor or major associations, respectively

### Nutritional and environmental factors

#### Acidic drinks

The consumption of acidic beverages—particularly fruit juices and soft drinks—has long been regarded as one of the most significant contributors to ETW in children and adolescents (Lussi and Carvalho 2014), because these drinks are often consumed in large volumes and contain acids (Hunter et al. 2009) like citric or phosphoric acid. In the last few years, soft drink consumption has markedly increased worldwide, especially among children and adolescents. A continuous upward trend has been observed in Europe (UNESDA 2024) and other countries (Euromonitor_International 2011), reaching staggering values of 300% increase in the United States over a period of 20 years (Cavadini et al. 2000).

The literature, however, shows mixed findings when associating dietary factors and ETW in children. While several studies report a significant association between dietary acids and ETW in children (Gatou and Mamai-Homata 2012; Harding et al. 2003; Luo et al. 2005; Murakami et al. 2011; Nayak et al. 2010), others find no clear correlation (Ayers et al. 2002; Rios et al. 2007; Warren et al. 2002; Wiegand et al. 2006). This inconsistency may derive from differences in study design, dietary assessment methods, or other confounding factors like oral hygiene. In any case, very young children are more frequently being exposed to such drinks (Fulgoni and Quann 2012; Williams et al. 2013) at a very young age, and this may strongly influence the early establishment of dietary habits that will persist through adolescence and adulthood (Lien et al. 2001).

Sports and energy drinks are also popular among adolescents, with consumption also rising globally. In the United States, the average person consumes around 29 litres of energy and sports drinks per year, with projections that this value may reach 30 L/person in the near future (Statista 2024). The U.S. currently leads the sports drink consumption, whereas Europe ranks highest in energy drinks (BSDA 2013), where 68% of adolescents have already consumed energy drinks at least once in the past year (Zucconi et al. 2013:EN-394). Alarmingly, even very young children (3–5 years) have already been exposed to energy drinks (around 2%), and this number increases with age, reaching values of 19% of 6–10-year-olds (Zucconi et al. 2013:EN-394). These drinks often combine high acidity with high sugar content, making them particularly harmful for oral health.

Besides acidic drinks, many candies, which are usually specifically marketed for children or adolescents, are acidic and can show a high erosive potential (Lussi et al. 2019b). Even presumably healthy products like vitamin gums are acidic and drop the pH of saliva to low values for a prolonged period of time (Gambon and Brand 2021).

#### Medications

Certain medications and supplements are made with acidic formulations, thereby increasing their chances of causing ETW. A meta-analysis showed that patients that make use of vitamin C supplementation—often as chewable or effervescent tablets—have 16% higher odds of developing ETW (Li et al. 2012). For children who use asthma medications (Al-Dlaigan et al. 2002; Dugmore and Rock 2003; Gurgel et al. 2011; Shaw et al. 2000), some studies suggest that they may cause ETW, while others find no association. However, most studies on which such observations are based usually use univariate analyses, and these findings must be interpreted cautiously. It is strongly advisable for future studies to use regression models that control for other confounders. Only in such multivariate models would the association (or lack thereof) be clearer.

#### Environmental and occupational exposure

In young children, environmental and occupational exposure to acids are generally considered negligible, but some factors may be more noteworthy for adolescents, specifically those involved in high-performance sports. Assiduous/professional athletes are exposed to several conditions that may increase the risk of ETW, like decreased salivary flow rate during extended physical exertion, frequent consumption of sports drinks for rehydration and electrolyte balance, and exposure to chlorinated swimming pools, specifically frequent swimmers. Some chlorinated swimming pools have pH levels as low as 3 (Abdelrahman et al. 2023). Prevalence of ETW in adolescent swimmers (12–17 years old) can be as high as 25% (Zebrauskas et al. 2014). Even children as young as 6–14 years who train regularly in swimming pools showed a significantly higher prevalence of ETW (Marqués Martinez et al. 2019), although it was not clear whether the wear was more severe on their primary or permanent teeth. Moreover, the risk of ETW increases with training duration, with adolescents training for over three years having more than five times the odds of developing ETW (Rao et al. 2019).

### Patient-related factors

#### Genetics

Among the genetic factors with the potential role in the development of ETW, gender has been the most studied. Evidence suggests that males are generally more susceptible to ETW than females (Marschner et al. 2024b), specifically in childhood (looking at primary teeth), where a systematic review identified eight studies, all of which showed that boys experienced significantly more ETW than girls (Kreulen et al. 2010). One factor leading to such a difference could be a difference in taste reception (Gambon et al. 2012), leading to different diet preferences. The gender disparity is, however, not corroborated in a more recent review (Marschner et al. 2024a). The specific genetic mechanisms for ETW are still not fully understood. It is possible that some biological and behavioral factors—like tooth formation (Vieira 2021c), saliva (Vieira 2021b), pellicle (Wiegand et al. 2019), diet preference (Vieira 2021d), or behaviour (Vieira 2021a)—can play a role, but more studies are still necessary.

#### Saliva and pellicle

Saliva and the salivary pellicle are important in protecting the teeth against ETW. There are several chemical differences between saliva from children and adults, suggesting a maturation in the fluid. This specifically affects the acquired pellicle, which has been shown to be much thinner on deciduous enamel—about one-third the thickness of that on permanent enamel (Sonju Clasen et al. 1997). The pellicles also differ in their proteomic composition, with pellicles formed on primary teeth sharing only 42% of the proteins with pellicles formed on permanent teeth (Zimmerman et al. 2013). In a systematic review on the role of salivary factors related to ETW (Madariaga et al. 2023), the authors indicated that salivary pH might play a significant role, but only 26 studies (from a total of 111) looked specifically at children and adolescents. Moreover, there was no separation for ETW on primary and permanent teeth, so it is highly probable that the results focus rather on the effect of adult saliva on permanent teeth, whereas the protective effect of children’s saliva on primary teeth has generally been neglected in research. Nonetheless, it can be inferred that saliva and pellicle have protective benefits against ETW. In young children, saliva provides better protection for primary teeth, which is slightly more problematic for older children during the mixed dentition phase as well as for adolescents, whose permanent teeth will not be as protected with the (non-mature) saliva.

#### Eating disorders and gastroesophageal reflux

Eating disorders (ED) and gastroesophageal reflux (GER) are two medical conditions that can greatly contribute to ETW. This has already been shown for adults, since EDs and GER can expose teeth to gastric acids, leading to enamel demineralization and wear. While EDs are more common in adolescents and adults, children can still be affected by the condition (Williams et al. 2010), though with very low prevalence indeed (0.003%) (Nicholls et al. 2011). Although EDs will not play a major role in ETW during childhood, GER is a more prevalent condition, affecting up to 86.9% of babies at two months of age (Osatakul et al. 2002), 24.4% of children by 2 years of age, and only 7.2% of children between ages 2 and 11 (Martigne et al. 2012). Despite the decreasing prevalence of GER during childhood, its association with ETW in this age group is still under debate. While some studies support a positive correlation (Aine et al. 1993; Dahshan et al. 2002; O'Sullivan et al. 1998) of GER and ETW in young children, others do not find any significant association between the two conditions (Ersin et al. 2006; Linnett et al. 2002; Wild et al. 2011). Two systematic reviews have also shown conflicting conclusions on this topic (Firouzei et al. 2011; Pace et al. 2008), while another showed a higher prevalence of ETW in children with GER (Lechien et al. 2020), but it did not distinguish between primary and permanent teeth. A possible explanation for this divergence is that reflux episodes in children may not last long enough to cause significant wear on primary teeth (Carvalho et al. 2014), or the protective effect of the child saliva on these teeth may interact postively against ETW. This may not be the case for older children and adolescents. In this older age group, the association is clearer. Carvalho et al (Carvalho et al. 2025) found 7 systematic reviews associating GERD and ETW in children/adolescents, the majority of which centered on adolescents, where a significant association was found between GER and ETW.

#### Behaviours and habits

The physical action and the time of contact between an acidic substance passing over the teeth can greatly affect the amount of demineralization and wear. Behaviour that prolongs the time drinks stay in contact with teeth—such as holding, swishing, or sucking drinks—can substantially enhance the risk of ETW. Interestingly, 43% of children who presented ETW also exhibited such habits (O'Sullivan and Curzon 2000). A systematic review also identified holding beverages in the mouth as a significant factor in ETW (Marschner et al. 2024a). It has also been suggested that if drinks were placed behind the teeth and swallowed, it could reduce the contact and, thus, reduce ETW. In this view, drinking from a straw in the correct way has been identified as a potential protective behavior (Marschner et al. 2024a), but the correct use of straws by young children might be rather the exception (Gambon 2011). Furthermore, feeding young children through baby bottles still remains contradictory. The prolonged use of bottles or feeding cups—especially beyond six months—has been shown to increase the risk for ETW (Ayers et al. 2002), especially if parents use these bottles to feed their children fruit juices (Luo et al. 2005). Moreover, timing of consumption seems to matter: for instance, giving children fruit juice or syrup in a bottle before bedtime significantly increases the risk for ETW. Most importantly, also for caries. So, it is clear that some eating and drinking behaviours may be associated with the development of ETW in younger children (Järvinen et al. 1991).

For adolescents, on the other hand, these factors are not of great importance, since no associations were found between ETW on permanent teeth and holding drinks in the mouth, or drinking from a straw, or toothbrushing related factors (Marschner et al. 2024b). Actually, under normal conditions, toothbrushing will not cause harmful effects to the tooth surface (Addy and Hunter 2003), and, in general, even after an acidic intake, toothbrushing will not lead to ETW (Bartlett et al. 2013; Fernández et al. 2024; O'Toole et al. 2017). All in all, when regarding eating habits, the major effect might be on younger children (primary teeth) rather than on adolescents.

Tooth grinding (bruxism) is another important risk factor, particularly during childhood (Tvilde et al. 2021). The impact of bruxism seems to increase with age, being more prominent in pre-adolescents (10–12 years old) (El Aidi et al. 2011), where most children have a mixed dentition. However, the studies do not differentiate whether bruxism in this age group is rather affecting the primary teeth (which have been longer in the mouth) or the newly erupting permanent teeth. In older children and adolescents, the link between bruxism and ETW becomes harder to define, largely due to co-existing factors that will also influence the condition (Li et al. 2018).

## The role of fluoride and stannous ions in prevention of dental erosion and ETW

Before implementing preventive strategies against dental erosion and erosive tooth wear (ETW), it is important that these measures are preceded by a thorough risk assessment. This should include a review of the etiological factors, as well as a detailed assessment of the patient’s current preventive measures. Besides education on the erosive potential of different drinks/foodstuffs and recommendations on possible changes in eating/drinking habits, a specific focus should be put on oral hygiene habits and fluoride use.

Even though the effect of fluoride against dental erosion has been questioned over the years, there is today strong evidence that it can both prevent its onset and slow its progression. During erosion, the tooth surface undergoes complete dissolution of the apatite minerals in both enamel and dentine, although the surface may still feel hard. Since remineralization involves the regrowth of previously existing apatite crystals, this process is generally not considered feasible in the context of erosion, where the original crystal structure has been entirely lost (Ganss et al. 2004; Turssi et al. 2004).

When it comes to the use of fluorides, it is important to consider both the type of fluoride compound and the method of administration. Considering its manifestation with a total breakdown of tooth substance in comparison to the demineralized tissue seen for the caries lesion, the use of higher fluoride concentrations and more frequent administration has been recommended for prevention of dental erosion compared to dental caries (Lussi et al. 2019a, b). Additionally, evidence suggests that certain fluoride sources may be more effective than others (Carvalho et al. 2015).

A way in which the fluoride ion can act in relation to erosion damage is the formation of CaF₂-like layers, particularly attainable when using higher fluoride concentrations. This can be achieved by different monovalent fluoride sources such as sodium fluoride (NaF), sodium monofluorophosphate (MFP) and amine fluoride (AmF) as well as polyvalent metal cations such as stannous fluoride (SnF_2_). Protection by the monovalent fluoride compounds has been found to have only a limited effect against dental erosion (Ganss et al. 2011; Hooper et al. 2007). The majority of these studies have been conducted in vitro, with only a few performed in situ, where the more complex environment including saliva and the acquired pellicle, offers a more realistic simulation of the clinic.

Another mechanism of action involves the use of polyvalent metal ions. These ions, through their binding to the surface, have the ability to form a metal-rich surface layer, which provides protection against acid-related challenges. Among the metal cations, tin (Sn) in the +2 oxidation state (Sn^2+^), when it is called stannous ion, has received increased interest. Today it is commonly used in different oral hygiene products, especially in the form of stannous fluoride (SnF_2_), and recommended to be favourable in relation to dental erosion. It is often used in its stabilized form which refers to the incorporation of chelating agents, such as gluconate, to enhance the bioavailability and chemical stability of stannous ions. Stannous ions derived from SnF_2_ interact with both enamel and dentine, leading to the formation of a metal-rich layer which is primarily composed of tin phosphates or tin oxide compounds. This layer serves as a physical barrier that decreases the solubility of enamel and dentine, which also is limiting the direct exposure of the underlaying mineralized tissue to dietary or gastric acids. Due to its relative resistance to acid, this protective layer is particularly effective in defending against repeated erosive attacks. Therefore, it is thought to function both as a protective barrier that helps reduce enamel and dentine demineralization and as a promotor of enhanced remineralization. Positive effects against dental erosion have been found when tested as a slurry both in vitro (Ganss et al. 2011) as well as in situ (Huysmans et al. 2011).

Various compound combinations and concentrations of stannous ions have been evaluated, yet none showed a distinctly stronger effect (Ganss et al. 2012). The possibility for an enhanced effect by a combination of stannous fluoride and an organic compound has been examined. For this, chitosan has been added, which is a cationic polysaccharide recognized for its ability to bind to enamel. An increased protection of enamel has been found by this combination (Ganss et al. 2012; Schlueter et al. 2013), while no such effect was seen for dentine (Ganss et al. 2014). A systematic review found that the use of SnF_2_-containing toothpaste results in significantly lower dissolution and surface roughness compared with controls (Konradsson et al. 2020). Furthermore, the use of mouthrinses or toothpastes containing stannous fluoride (SnF_2_) or stannous chloride (ClF_2_) has been suggested to slow down progression of erosive tooth wear in a consensus report from the European Federation of Conservative Dentistry (Carvalho et al. 2015). In conclusion, stannous fluoride provides a broad-spectrum defence against erosive tooth wear by combining chemical reinforcement and physical protection of tooth surfaces. Its dual mechanism of action makes it especially useful in clinical strategies for managing patients who are at risk for, or are already experiencing, dental erosion and ETW.

An effect has not only been observed on the protection of tooth hard tissues. Although more relevant to caries, the antimicrobial properties of fluoride can also help manage biofilm activity. Stabilized stannous fluoride dentifrice has also been found to reduce the levels of gingivitis and gingival bleeding (Biesbrock et al. 2019; Clark-Perry and Levin 2020).

Among other polyvalent fluoride sources, titanium (Ti^4+^) has been suggested to have the capacity to offer protection against dental erosion (Lussi and Carvalho 2015; Sakae et al. 2024). A meta-analysis found strong evidence supporting that both stannous fluoride and titanium tetrafluoride agents may be effective in preventing erosions (da Silva et al. 2022). However, stannous ion-containing formulations are suggested to be more closely linked to minimizing enamel surface loss than promoting the rehardening of previously softened enamel (Lussi and Carvalho 2015).

Another approach has been to use high-fluoridated toothpastes containing NaF. This is a strategy which has been found promising for prevention of dental caries, and an improved protection of enamel against dental erosion has also been found for 5000 ppm F both in vitro (Moretto et al. 2010) as well as in situ (Ren et al. 2011) when compared to 1100 respective 1450 ppm F. A similar effect has not been proven for dentine (Magalhães et al. 2008).

The use of toothpaste is a fundamental component of preventive dental care. However, when it comes to dental erosion, certain concerns arise both regarding toothpaste formulation as well as brushing habits. Research has demonstrated that previously eroded enamel and dentine can suffer additional damage, not only as a result of the abrasiveness and dilution rate of the toothpaste (Turssi et al. 2010), but also due to mechanical forces during brushing (Wiegand et al. 2007). It is therefore essential to reach a balance—achieving effective cleaning through abrasive particles while avoiding further harm to the dental tissues. The presence of fluoride may help modulating this balance, reducing the overall abrasion through its effects.

For individuals at increased risk of dental erosion, it is important to complement twice-daily use of fluoridated toothpaste with additional preventive measures. A wide range of strategies is available to enhance fluoride exposure, including the use of mouth rinses, gels and varnishes. Also from this perspective, polyvalent metal ions have been found to exhibit a better efficacy in preventing erosion and ETW when used in rinse solutions. So has a stannous fluoride mouthrinse shown added benefits in vitro studies when used in combination with an anti-erosion toothpaste (Jiemkim et al. 2023). While stannous ion-containing solutions have shown some positive effects, the overall findings remain inconclusive, with differing impacts observed between deciduous and permanent enamel (Lussi et al. 2019a, b).

Few negative effects have been reported with the use of polyvalent metal ions, though they may cause mild extrinsic staining and taste disturbances. From a manufacturer´s perspective it is known that stannous fluoride is less stable compared to sodium fluoride and requires proper formulation to maintain efficacy.

Preventive strategies for the onset as well as further progression of erosion and erosive tooth wear (ETW) should always include fluoride, with particular focus on administering it in combination with polyvalent metal ions such as stannous fluoride. However, given the multifactorial etiology, it is also essential to try to reduce its etiological factors as well as incorporate other complementary preventive approaches.

Toothpastes and solutions are rarely tested regarding their erosion-protective effect on deciduous teeth. There are formulations specifically marketed for children, but children from 6 years on can also already use adult toothpastes with 1450 ppm F, and they already present a mixed dentition. When a range of anti-erosion toothpastes was tested both on deciduous and permanent teeth, normal NaF containing toothpastes could not reduce ETW on deciduous teeth. But, remarkably, the stannous ion-containing toothpaste, as well as an NaF containing toothpaste specifically marketed for children, both provided some protection for the deciduous teeth against ETW (Assuncao et al. 2019; Assuncao et al. 2018). Additional studies should be carried out to elucidate further how to prevent ETW in children, focusing on anti-erosion products specifically marketed for children. For more clinical relevance, future study-models should also include the use of children’s saliva, as it interacts with and protects the teeth differently from adult’s saliva (Carvalho et al. 2016).

## Future possibilities: acid-protective layers

Any layer restricting contact between erosive acids and mineralized tooth surfaces can help prevent dental erosion and ETW. Approaches include the use of sealants and film-forming polymers, modification of the acquired pellicle (AP) and preservation of the dentine organic matrix.

Sealants, adhesives, or flowable composites have been proposed for protection, with coating thickness ranging from a few micrometres (adhesives) to over 100 µm (flowable composites) (Tereza et al. 2016; Wegehaupt et al. 2017; Zhao et al. 2016; Zhao et al. 2017). Because bond strength to eroded dentine is reduced, pre-treatment to remove or stabilize the demineralized collagen layer is required before resin application (Wiegand et al. 2021). *In vitro* studies show that adhesives, sealants and flowable composites prevent enamel and dentine loss (Oliveira et al. 2015; Wegehaupt et al. 2013; Wegehaupt et al. 2012), while resin infiltrants demand application in excess (Oliveira et al. 2015; Tereza et al. 2016). *In situ* and clinical data indicate that protection lasts 3–9 months, depending on material and thickness, with sealants outperforming bonding agents (Bartlett et al. 2011; Sundaram et al. 2007). After 12 months, sealed teeth exhibited more wear than controls, attributed to fracture of sealant remnants and loss of underlying tissue. Thus, frequent re-sealing is required for clinical relevance (Azzopardi et al. 2004). Development of improved materials is necessary to achieve long-lasting effects.

Most studies on film-forming polymers have tested whether modifying acidic drinks with ovalbumin, casein, pectin, alginate or linear sodium polyphosphate reduces their erosive potential (Barbour et al. 2005; Beyer et al. 2010; Kairalla et al. 2025). Fewer examined incorporation into toothpastes or rinses. Chitosan has drawn interest for adsorbing onto saliva-coated hydroxyapatite, forming a hydrophobic layer (Lee et al. 2012; van der Mei et al. 2007) that resists citric acid, though not completely (Lee et al. 2012). Its efficacy depends on concentration and exposure time (Arnaud et al. 2010). *In vitro*, chitosan toothpastes without fluoride prevented erosion comparably to conventional fluoride formulations (Ganss et al. 2011). Combined with stannous fluoride, chitosan enhanced protection *in vitro* (Ganss et al. 2012) and *in situ* (Schlueter et al. 2014), though data on sodium fluoride is lacking. Mechanisms may involve multilayer formation and electrostatic interactions with enamel, pellicle, and abrasives (Schlueter et al. 2014). Other polymers—poly(alkylmethacrylate)s (Nielsen et al. 2011), fluoropolymers (Churchley et al. 2008), and systems with carboxymethylcellulose, copovidone and xanthan gum (Gracia et al. 2010)—showed partial protection, but were less effective than aqueous sodium fluoride (Churchley et al. 2008; Nielsen et al. 2011). Some, such as carbopol (Avila et al. 2020) and aminomethacrylate copolymer (Augusto et al. 2021a), may potentiate fluoride, while hydroxypropylmethylcellulose and polyoxirane reduce its effect (Augusto et al. 2021b). Despite promising laboratory results, clinical validation is needed (Augusto et al. 2022).

Upon acid exposure, the outer AP layer (globular) is removed, while the basal layer remains intact, suggesting greater protective role of the latter (Hannig et al. 2009). APs formed in 1 min protect as effectively as those formed in 2 h (Ventura et al. 2023). Proteomic tools identified acid-resistant proteins for potential use in “acquired pellicle engineering”: cystatin B against citric acid (Delecrode et al. 2015), hemoglobin (Martini et al. 2019) and statherin (Taira et al. 2018) against hydrochloric acid.

Recombinant cystatin CaneCPI-5 shows strong enamel binding (Santiago et al. 2017) and protects against erosion and erosion–abrasion *in situ* (Pela et al. 2022; Pela et al. 2021) and against erosion *in vivo* (Carvalho et al. 2020; Pela et al. 2023), when applied after prophylaxis. Vitamin E has been suggested as a carrier, delivering CaneCPI-5 into the basal pellicle without prophylaxis; both agents, applied separately (in distinct solutions), protected against initial erosion (de Oliveira et al. 2023). Further research is required to test their impact on advanced erosion and define clinical protocols. A single vehicle combining CaneCPI-5 and vitamin E, such as a gel, could simplify use.

Interestingly, CaneCPI-5 protects mainly against extrinsic erosion. To also target intrinsic challenges, a recombinant protein, CaneStat, was created by fusing CaneCPI-5 with the N-terminal 15 amino acids of statherin, where phosphorylated serines were replaced by aspartic acid (phosphomimetic peptide). Initial *in vitro* data showed protection against both intrinsic and extrinsic erosion (Ferrari et al. 2025), but evaluation under clinically relevant conditions is still needed.

Other molecules, such as vegetable oils (Ionta et al. 2017) and polyphenols, including resveratrol, green tea extract (epigallocathechin galate), proanthocyanidin, grape seed extract, tannic acid and cranberry extract may also enhance AP protection, potentially reducing erosion (Baumann et al. 2023; Hertel et al. 2017; Mailart et al. 2025; Niemeyer et al. 2021; Niemeyer et al. 2023; Reis et al. 2024a; Reis et al. 2024b; Schestakow et al. 2024). Polyphenols have a dual effect (Niemeyer et al. 2023): modifying dentine by inhibiting proteases (Buzalaf et al. 2015) or promoting collagen crosslinks (Schestakow et al. 2023), thereby reducing the degradation of the dentine demineralized matrix, and directly altering AP composition and structure. After polyphenol exposure, the AP becomes thicker and more electron-dense, with increased acid-resistant proteins (de Souza et al. 2017; Levy et al. 2024; Pela et al. 2019; Rehage et al. 2017; Reis et al. 2024a; Schestakow et al. 2022; Schestakow et al. 2024; Silva et al. 2021; Zimmermann et al. 2019), enhancing protection. However, as with other actives, clinical validation for polyphenols is lacking.

## Take-away points/conclusion

The following take-way points can be drawn from the current evidence. These points are also summarized in Table 2.

- Primary enamel is more susceptible than permanent enamel in more severe cases.
- Nutritional factors are the main culprit in ETW for children and adolescent. As the children get older, there seems to be sufficient evidence connecting acidic dietary factors to ETW in permanent teeth.
- Saliva (and pellicle) is a major protecting factor for children and adults alike.
- Gastro-oesophageal reflux (GER) may have some association with ETW in primary teeth, but it is of much greater importance for older children and adolescents.
- Brushing by itself does not cause any wear. Only in association with erosion (surface softening), it can contribute to ETW.
- For dental erosion and thus ETW, there is no general critical pH, as it depends on the specific composition of the erosive substance.
- Fluoride and polyvalent metal ions such as stannous ions in dental hygiene products help reducing ETW.
- Promising future preventive methods include the use of polymers, proteins, and polyphenols in dental hygiene products.

## Data Availability

No datasets were generated or analysed during the current study.
